# Kynurenines and Inflammation: A Remarkable Axis for Multiple Sclerosis Treatment

**DOI:** 10.3390/ph17080983

**Published:** 2024-07-25

**Authors:** Paul Carrillo-Mora, Carlos Landa-Solís, David Valle-Garcia, Alexandra Luna-Angulo, Hamlet Avilés-Arnaut, Benjamín Robles-Bañuelos, Laura Sánchez-Chapul, Edgar Rangel-López

**Affiliations:** 1 Clinical Neurosciences Division, National Institute of Rehabilitation “Luis Guillermo Ibarra Ibarra”, Mexico City 14389, Mexico; pcarrillo@inr.gob.mx; 2 Tissue Engineering, Cell Therapy, and Regenerative Medicine Unit, National Institute of Rehabilitation “Luis Guillermo Ibarra Ibarra”, Mexico City 14389, Mexico; clanda@inr.gob.mx; 3 Neuroimmunology Laboratory, National Institute of Neurology and Neurosurgery “Manuel Velasco Suárez”, Mexico City 14269, Mexico; david.valle.edu@gmail.com; 4 Neuromuscular Diseases Laboratory, Clinical Neurosciences Division, National Institute of Rehabilitation “Luis Guillermo Ibarra Ibarra”, Mexico City 14389, Mexico; abluna@inr.gob.mx; 5 Faculty of Biological Sciences, Institute of Biotechnology, National Autonomous University of Nuevo Leon, Nuevo León 66455, Mexico; hamlet.avilesarn@uanl.edu.mx; 6 Cell Reprogramming Laboratory, National Institute of Neurology and Neurosurgery “Manuel Velasco Suárez”, Mexico City 14269, Mexico; robles0610@outlook.es

**Keywords:** multiple sclerosis, inflammation, kynurenines pathway, kynurenine metabolites, quinolinic acid, treatment

## Abstract

Multiple sclerosis (MS) is a chronic inflammatory autoimmune neurological disease characterized by the recurrent appearance of demyelinating lesions and progressive disability. Currently, there are multiple disease-modifying treatments, however, there is a significant need to develop new therapeutic targets, especially for the progressive forms of the disease. This review article provides an overview of the most recent studies aimed at understanding the inflammatory processes that are activated in response to the accumulation of kynurenine pathway (KP) metabolites, which exacerbate an imbalance between immune system cells (e.g., Th1, Th2, and T reg) and promote the release of pro-inflammatory interleukins that modulate different mechanisms: membrane-receptors function; nuclear factors expression; and cellular signals. Together, these alterations trigger cell death mechanisms in brain cells and promote neuron loss and axon demyelination. This hypothesis could represent a remarkable approach for disease-modifying therapies for MS. Here, we also provide a perspective on the repositioning of some already approved drugs involved in other signaling pathways, which could represent new therapeutic strategies for MS treatment.

## 1. Introduction

Multiple sclerosis (MS) is a chronic central nervous system (CNS) inflammatory disease of autoimmune origin. Although the most important target of initial damage in MS is brain and spinal cord white matter-myelin, the functionality of axons and glial cells are also involved in different phases of the disease [1]. Clinically, MS is initially characterized by periods of exacerbation and remission, but over time, the disease shows a progressive and neurodegenerative behavior that is generally associated with different levels of disability and a decrease in the quality of life of affected patients [2]. MS is one of the most important causes of chronic disability worldwide [3]. The precise origin of the disease is not known, but it is proposed that there is a complex combination of genetic, geographic, environmental, and even infectious factors [4].

Since the introduction of interferon beta 1a for the treatment of MS in 1993, there was a very important development of new pharmacological therapies that, due to their clinical effectiveness, can currently be considered as true disease-modifying therapies [5]. However, the disease was shown to be largely pleiotropic and dynamic. So, there is a continued need for the development of new treatment strategies, particularly for progressive forms of the disease where most currently available therapies are not effective [6].

In this sense, the possible participation of kynurenines in the pathophysiology of MS was investigated for many decades. The first clinical observation that related the possible participation of the kynurenine pathway (KP) in MS was that reduced levels of tryptophan were observed both in blood and in cerebrospinal fluid (CSF), which implied that there was greater activation of KP in the CNS of these patients [7]. As a result of this seminal observation, a great diversity of clinical studies demonstrated different changes in kynurenine levels in patients with MS, both in blood and in CSF [8]. However, beyond only demonstrating the participation of kynurenines in the pathophysiology of MS, the importance of their study lies in the fact that they may also represent a new therapeutic target with multiple potential actions.

This review article pretends to highlight information about the involvement of KP metabolites in the initiation and maintenance of the chronicity of the typical inflammatory response observed in MS. Therefore, we propose that modulation of KP may represent a therapeutic target aimed at decreasing the accumulation of KP metabolites in the CNS that exacerbates an imbalance between immune cells, triggering cell death mechanisms and promoting neuronal loss and axon demyelination. In addition, we also provide a perspective on the repositioning of some already approved drugs for other diseases that could represent new therapeutic strategies for the treatment of MS patients.

## 2. Epidemiology

According to the Global MS Atlas, the number of patients worldwide living with MS increased continuously in recent decades, resulting in a global prevalence of 35.9 per 100,000 inhabitants [9]. The average age of onset of the disease is between 20 and 30 years of age, and most studies reported a clear predominance of the disease in females [10]. This increase in the disease incidence seems to be explained—at least partially—by an increase in disease diagnosis and surveillance. It is interesting to highlight, that despite this incidence increase, a reduction in the rates of disease progression and disability, as well as an increase in life expectancy, were also demonstrated, which may be related to the recent development and extended use of highly effective disease-modifying therapies [11]. Regarding ethnicity, it was observed that Caucasians are usually the most affected, followed by African-Americans, who have similar prevalence, followed by Hispanics and Asians [12].

## 3. Clinical Features

Demyelinating lesions in MS can appear anywhere in the CNS white matter, however, the most frequent clinical manifestations are s optic neuritis, partial spinal cord syndromes, sensory syndromes, and cerebellar involvement [2]. More than 85% of patients initially present a disease behavior with periods of exacerbation (attacks or outbreak) and periods of remission of symptoms (relapsing–remitting clinical variety). Only a 5 to 15% of patients show from the beginning a slowly progressive behavior without presenting clear symptom remission periods (primary progressive clinical variety). In patients with the relapsing–remitting clinical variety, they begin to present fewer and fewer “attacks” and show a slow progressive evolution after 10–15 years of disease evolution (secondary progressive clinical variety) [13,14]. This clinical behavior led to the proposal that the disease in its initial stage has a predominant inflammatory component, and in the progressive stage, a predominant degenerative component, where a slowly progressive functional and cognitive deterioration is observed, which coincides with some brain structural markers, such as brain atrophy [15]. These clinical varieties are especially relevant because most of the disease-modifying treatments available today are indicated for relapsing–remitting types, while progressive or degenerative forms of MS have very few therapeutic options that show significant effects [14].

## 4. Diagnosis

Diagnosis of MS requires a combination of clinical information, neuroimaging, and laboratory studies. Although diagnostic criteria continue to evolve over time, diagnosis of MS essentially relies on demonstrating that the patient’s demyelinating brain lesions meet the criteria for spatial and temporal dissemination. Spatial dissemination criteria mean that the patient has lesions at different anatomic sites, for example, cortical or juxtacortical lesions, periventricular lesions, and lesions in the brainstem, optic nerve, or spinal cord. Such lesions may be evidenced either clinically (clinical symptoms and signs) or by neuroimaging studies, or, ideally, by both methods [16]. On the other hand, dissemination in time criteria requires demonstrating the appearance of new demyelinating lesions where none existed before. This can be documented by the presence of a new attack or clinical relapse, or also by showing in magnetic resonance studies that there are lesions at different temporal stages; for example, the coexistence of chronic lesions without acute inflammation and recent or new lesions that present acute inflammation (gadolinium enhancement). In patients in whom the dissemination over time criterion is not met, the presence of oligoclonal bands in the CSF may be sufficient to meet this criterion. The latest revision of the diagnostic criteria in MS was updated in 2017, and it can be consulted from various sources [16]. Finally, it is very important to highlight that the established diagnostic criteria are not infallible and were not designed to make a differential diagnosis between MS and other similar pathologies. So, the differential diagnosis must always be carried out in order to rule out other inflammatory, infectious, metabolic, deficient, toxic, or systemic pathologies, especially in patients with atypical characteristics [17].

## 5. Treatment

To date, there is no curative treatment for MS. For several decades, there was a great drug development to try to reduce the rate of clinical relapses in MS patients. The current efficacy of these therapies led them to be considered as true disease-modifying therapies, since they were shown to significantly reduce the annualized clinical relapse rate as well as secondary disability [11]. Currently, there are approximately 14 disease-modifying drugs approved by the Federal Drug Administration (FDA) for the treatment of the relapsing–remitting variety of MS: 2 interferons, 3 generic immunomodulators (glatiramer acetate, teriflunomide, and dimethyl fumarate); 4 sphingosine phosphate modulators; and 5 monoclonal antibodies [18]. There are several ways to classify them, depending on their mechanism of action, but one of the most practical classification ways is according to their therapeutic efficacy [19]. They are generally divided into two main groups: drugs of moderate efficacy, that is, those that reduce the annualized relapse rate by up to 30% (e.g., interferons, teriflunomide, and glatiramer acetate); and high-efficacy drugs, for those that reduce the relapse rate by more than 50% (e.g., natalizumab, alemtuzumab, cladribine, etc.) [19]. However, at present, the optimal treatment of progressive forms, whether primary progressive or secondary progressive, is poorly defined and remains controversial due to its limited clinical efficacy [20]. It is evident that brain damage mechanisms are completely different in these MS progressive phases. So, there is still debate about which should be the ideal therapeutic targets in these degenerative forms of the disease. In this regard, some authors suggest that the drugs used in this phase should combine different mechanisms of action: anti-inflammatory effects, especially on microglia, lymphocytes and neutrophils, reduction in oxidative stress generated by iron deposition, improvement of mitochondrial dysfunction and, also, the promotion of remyelination [21].

## 6. Cellular Dysfunctions Induced by MS

### 6.1. Loss of Myelin Sheet on Axons

MS is characterized by the destruction of the myelin basic protein, one of the most important proteins in the membrane that covers axons. This leads to damage to axon conformation and neuronal cell bodies, as demonstrated by MRI studies, thus leading to disturbances in nerve communication, resulting in a loss of physical and mental abilities. Some in vivo studies demonstrated that fibroblast growth factor 2 (FGF2) exerted negative effects on the morphology and functions of mature oligodendrocytes and significant loss of myelin [22]. FGF2 also induces changes in the immunoreactivity of Tau protein, which is associated with the production of myelin membranes by oligodendrocytes. The increased accumulation of Tau, mediated by FGF2, eventually leads to an immune attack against these myelin-forming oligodendrocytes and to abnormal axonal myelin sheath formation with consequent loss of nerve transmission [23]. Some immune responses triggered by abnormalities in B lymphocyte activity are also involved in the attack on myelin sheath, as they produce antibodies against the myelin structure that contribute to the loss of neuronal activity and the pathogenesis and progression of some demyelinating and neurodegenerative diseases such as MS [24].

### 6.2. Macrophage Responses

Macrophages are central cells that are also involved in MS progression, along with other cells and molecules, such as activated T cells (secretors of interleukins), microglial cells, astrocytes, and molecules that mediate inflammation: anti-myelin antibodies, tumor necrosis factor alpha (TNF-α), interferon gamma (IFN-γ), and serum complement. Together, these are involved in the myelination process [25], but also upregulate the production of indoleamine 2,3-dioxygenase (IDO), a key player in KP [26]. In addition, demyelinated tissue was detected in focal lesions of MS samples [27], and apoptotic signatures were clearly observed in neurons, leukocytes, and oligodendrocytes in these areas [27]. T cell and macrophage presence, which are associated with myelin degradation in MS, were also observed at these sites [28].

In experimental animal models of MS, macrophages were identified as the main contributors to the significant level increase in the tryptophan metabolites quinolinic acid (QA) and 3-hydroxykynurenine (3-HK), which in combination act as two neurotoxic metabolites that have the ability to be deposited in the CNS of rats [29]. On the other hand, neopterin is a protein released by macrophages previously stimulated with IFN-γ, which in turn can modulate the expression of IDO [30]. Therefore, IDO, QA, and neopterin were considered as neurodegenerative biomarkers [31].

Experimental models demonstrated the immunomodulatory role of IDO in conjunction with IFN-γ, which is secreted by encephalitic T helper 1 (Th1) cells during infections in brain tissue. These studies show that IDO delays T cell proliferation and induces IFN-γ secretion by Th1 cells, which, together with infiltrating macrophages/activated microglia in the brain, are involved in the production of IDO, thus initiating a feedback loop that negatively modulates autoimmune inflammation in MS [32]. Based on these findings, some endogenous immunomodulators, such as peroxisome proliferator-activated receptors (PPARγ), PPARγ coactivator-1alpha (PGC-1α), and some metabolites of the KP, were explored as modulator molecules in MS.

### 6.3. Induction of Inflammatory Cytokines

Once macrophages and microglia are activated, proinflammatory cytokines are released in the blood and brain, leading to an increase in nitric oxide (NO) and prostaglandin E2 levels. This process triggers a defense mechanism to maintain homeostasis, which consists of the hypersecretion of cortisol to inhibit the synthesis of proteins and neurotrophic factors and prevent damage to neuronal networks. However, in MS progression and other pathologies associated with neurodegeneration, this protective response is not sufficient, as the induced secretion of proinflammatory cytokines is accompanied by the formation of neurotoxic end products from the tryptophan–kynurenine pathway, such as QA, which accumulates in astrocytes and neurons and causes dysfunction of neuroprotective and neuronal repair mechanisms, leading to disease progression [33].

Several subtypes of immune cells that modulate the secretion of proinflammatory cytokines, such as CD8+, CD25+, and FoxP3+ lymphocytes, are involved in MS development. These cells are able to modulate the function of autoreactive CD4+ T cells during the course of MS by downregulating the expression of co-stimulatory molecules in dendritic cells via a STAT3-mediated pathway and cytotoxic T lymphocyte antigen 4-dependent mechanisms [34,35]. Interleukin-6 (IL-6) is the proinflammatory molecule mainly associated with MS [36], followed by IL-1β [37], TNF-α, and TNF-γ [38]. These molecules are involved in the regulation of tryptophan/kynurenine metabolites, which are closely linked to glutamatergic neurotransmission. In particular, 3-HK and QA were identified as important metabolites that can induce neurotoxic and chronic effects in this disease [39].

**Figure 1:**
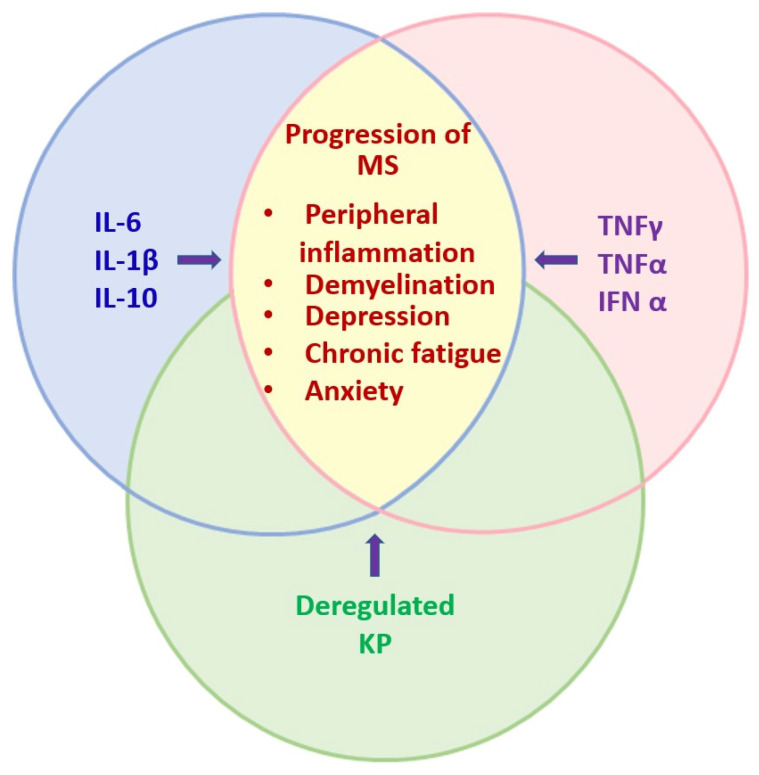
Synergistic effects of inflammatory cytokines in MS progression. The figure shows the interplay of several cytokines secreted by T lymphocytes, such as IL-6, IL-1β, and IL-10, which synergize with TNF-α, TNF-γ, and IFN-α to activate IDO and directly affect the metabolic pathways regulating tryptophan degradation products, leading to various neurological disorders, such as peripheral inflammation, demyelination, depression, chronic fatigue, and anxiety during MS progression.

The synergy of the inflammatory cytokines IL 6, IL-1β, TNF-α, and TNF-γ leads to IDO activation [40,41,42,43]. Activation of this immuno-inflammatory pathway leads to hyper- or hypofunction of tryptophan catabolites [43], which are associated with some neurological disorders [43], such as depression, anxiety, peripheral inflammation, and chronic fatigue [44,45,46,47,48], that are considered key factors in MS progression and patient quality of life [49] (Figure 1).

## 7. Kynurenines

### 7.1. Tryptophan Metabolism

Tryptophan is mainly degraded via the KP producing physiologically active metabolites, known as kynurenines (KYN), culminating in the formation of nicotinamide adenine dinucleotide (NAD^+^), which is an important cellular energy source [50]. In addition, KP also plays an important role in the activation of immune responses, inflammatory mechanisms, and intracellular processes involved in neurotransmission [51]. KP is present in various immune cells, such as dendritic cells, monocytes, and macrophages, and in some tissues, such as the liver [52]. However, not all human CNS cells contain all enzymes involved in KP. For example, it is present in oligodendrocytes, which do not contain the enzymes IDO-1 and IDO-2 and tryptophan dioxygenase (TDO). Previously, it was accepted that astrocytes lack of the enzyme kynurenine-3 mono oxygenase (KMO) involved in the metabolism of KYN to produce 3-HK. Recently, KMO was detected in these cells, but this enzyme is not active [53]. Therefore, astrocytes cannot produce the neurotoxic metabolite QA. In contrast, infiltrating macrophages, neurons, and activated microglia have the complete and functional KP [26,54]. Several reports demonstrated that the imbalance in the formation of these metabolites is associated with neurodegenerative mechanisms and some neurological disorders and psychiatric diseases, such as depression and schizophrenia [55,56].

**Figure 2:**
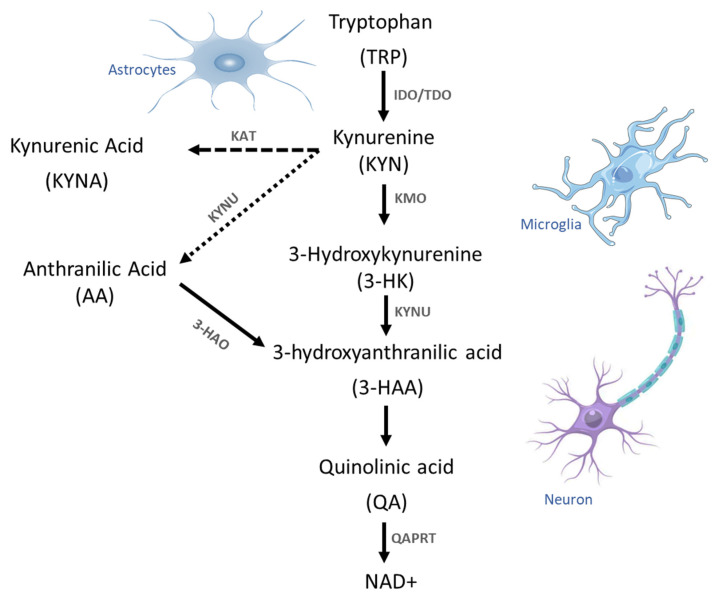
Kynurenine pathway in the CNS. Tryptophan is metabolized by the KP, whereby kynurenines are formed and finally NAD^+^ is produced. KP is divided into three main branches: in the first branch, KYN is metabolized into 3-HK by KMO and converted into 3-HAA by KYNU and into QA to finally produce NAD^+^. In the second branch, KYN is converted into the neuroprotective KYNA by KAT enzymes and in the third branch, KYN is converted into AA by KYNU. The complete and functional KP is present in infiltrating macrophages, neurons, and activated microglia, but not in astrocytes.

The first KP step is the conversion of TRP to KYN involving the enzyme TDO, which is localized in hepatocytes, and the enzymes IDO-1 and IDO-2, which are localized in various human tissues [55]. Under physiological conditions, KP is divided into three major branches. In the first branch, KYN is preferentially converted to 3-HK by KMO, which is converted into hydroxy anthranilic acid (3-HAA) by the enzyme kynureninase (KYNU), which is also involved in the biosynthesis of NAD cofactors from TRP. Additionally, 3-HAA is converted to QA by the cytosolic protein 3-hydroxyanthranilate 3,4-dioxygenase (HAAO). High QA concentrations exert excitotoxic effects, as QA is able to activate glutamate N-methyl-D-aspartate receptors. The activity of the enzyme quinolinic acid phosphoribosyl transferase (QAPRT) is the only pathway for QA metabolism that produces NAD^+^ and 5-phosphoribosyl-1 pyrophosphate [56]. In the second branch, KYN is converted into the neuroprotective kynurenic acid (KYNA) by the kynurenine aminotransferases (KAT) via an irreversible transamination step, whereby the KAT II isoform expressed in astrocytes is responsible for KYNA synthesis in the human brain. In the third KP branch, KYN is finally converted into anthranilic acid (AA) by KYNU (Figure 2). All these metabolites have neuroprotective, antioxidant, and anti-inflammatory properties, while others have opposite effects and exhibit neurotoxic, pro-oxidant, and pro-inflammatory properties [57]. Moreover, these molecules can regulate neuronal plasticity and excitability by modulating the activity of glutamate receptors and G protein-coupled receptor 35 (GPR35) in the CNS. In addition, these metabolites are also involved in determining the immune cell profile via the aryl hydrocarbon receptor (AHR), as an imbalance between neuroprotective and neurotoxic kynurenines occurs during inflammation, as suggested by the observed KYN/TRP ratio, which is considered a marker for activation of the cellular immune response [58].

It was reported that TRP, KYN, and 3-HK can cross the blood brain barrier (BBB) through the protein large neutral amino acid transporter (LAT1) [59], but in particular, about 60–80% of the KYN brain content may be of exogenous origin, although KYN can be formed from TRP during inflammation in this tissue [60]. KYNA cannot cross the BBB because it is synthesized almost exclusively in the CNS, and is a competitive antagonist of ionotropic glutamate receptors N-methyl-D-aspartate (NMDA), kainite, and α-amino-3-hydroxy-5-methyl-4-isoxazolepropionic acid (AMPA). When bound to its receptors, KYNA can inhibit excessive Ca^2+^ influx into neurons and also inhibit presynaptic α7-nicotinic acetylcholine receptors that regulate presynaptic glutamate release, exerting a protective effect on brain cells from death pathways even at low concentrations [61]. It is noteworthy that KYNA activity not only reduces the excitotoxic effect induced by QA, but also exhibits antioxidant activity that protects cells from lipid peroxidation and scavenges reactive oxygen species, demonstrating its neuroprotective effect [62].

QA is a specific competitive agonist of NMDA receptors that is involved in energy homeostasis under physiological conditions at nanomolar concentrations. However, under pathological conditions leading to chronic inflammation, as it occurs in MS, QA production is increased and it exhibits potent neuroexcitatory activity by enhancing glutamate excitotoxicity through increased Ca^2+^ influx, leading to cell death of astrocytes, oligodendrocytes, and neurons. In addition, QA increases ROS production [61,63], and inhibits the reuptake of glutamate by astrocytes [64]. The observed toxicity of QA induced at various concentrations in the CNS is due to its NMDA receptor sensitivity through the subunits NR2A, NR2B, and NR2C, which are mainly expressed in the striatum and hippocampus [65,66]. Furthermore, QA shows a synergistic neurotoxic effect with 3-HK, which significantly increases the extent of damage leading to the appearance of lesioned areas [67].

Several studies showed that 3-HK is a NMDA receptor-independent molecule produced mainly by microglia, and that its neurotoxic properties are associated with cell damage due to increased oxidative stress caused by the increased formation of ROS [68,69], leading to protein modification, lipid peroxidation, DNA damage, modulation in the inflammatory response, and finally cell death [69]. Studies in rats with experimental autoimmune encephalomyelitis, an animal model used for the investigation of MS, showed increased 3-HK levels in plasma, brain, and spinal cord [29]. Elevated 3-HK levels were also found in samples from MS, Parkinson’s, and Huntington’s disease patients [70], furthermore, both KYN and KYNA levels are slightly decreased in Parkinson’s patients. The immune response induced by QUIN in the surrounding of amyloid plaques leads to elevated excitotoxicity and oxidative stress in Alzheimer disease; also, it was demonstrated that QUIN induces the tau phosphorylation in cultured primary neurons [43].

### 7.2. Dysregulated Generation of Kynurenines

**Figure 3:**
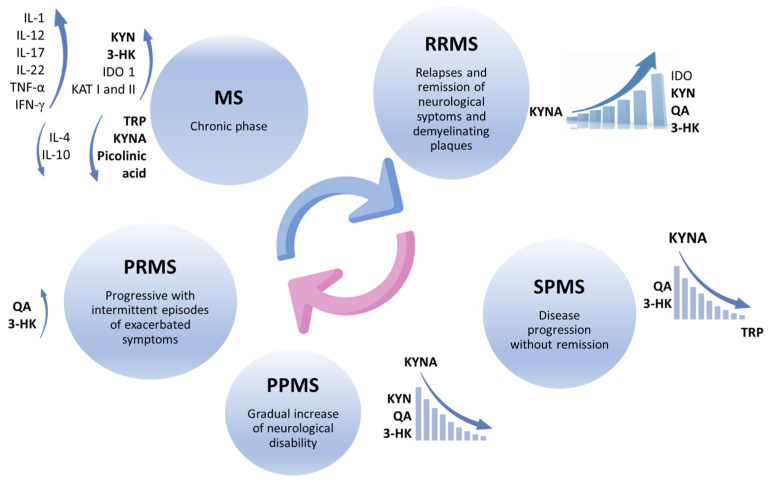
Changes in kynurenine metabolites in different phases of multiple sclerosis. KP activation by proinflammatory cytokines promotes the activation of IDO, KMO, and KAT and leads to high KYN and 3-HK levels in the chronic phase of MS, while levels of TRP, KYNA, and PA decrease. In RRMS, there is an increase in the expression of IDO-1, with a corresponding increase in KYN, QA, and 3-HK in serum, and both low and high concentrations of KYNA are found in patient’s CSF. In SPMS, TRP and KYNA levels are lower, and in PPMS, KYN, QA, and 3-HK are increased, while KYNA decreases. Finally, in PRMS, QA and 3-HK levels are high, contributing to neurodegeneration.

Alterations in the KP represent critical factors during MS pathogenesis, which may induce an imbalance between neuroprotective and neurotoxic metabolites produced in this signaling pathway, eliciting the appearance of neurotoxic effects and contributing to the development of neurodegenerative processes reported in progressive MS subtypes [30,71] (Figure 3). The KP is triggered by the activation of proinflammatory cascades, mainly induced by TNF-α and IFN-γ, which in turn activate the enzymes IDO and KMO, leading to changes in kynurenine levels. These disturbances in TRP metabolism inhibit the regulation of T cell activity [72,73]. Although activation of some KP enzymes decreases T cell proliferation and immunosuppression in the short term, chronic activation of the KP leads to the production of some neurotoxic metabolites, such as QA, which damage oligodendrocytes and alter the innate repair mechanism of remyelination [70]. It was reported that the concentrations of some KP enzymes, such as KAT I and KAT II, are higher in the red blood cells of MS patients than in control subjects [63].

However, some discrepancies were found with respect to kynurenine levels in blood and CSF. Some studies demonstrated low TRP levels in the blood and CSF of patients in the chronic phase of MS, confirming KP activation [74], while patients with relapsing–remitting MS (RRMS) were found to have increased IDO-1 expression with a corresponding increase in serum KYN and N-formyl kynurenine levels [30,75]. In contrast, other studies reported low and high levels of KYNA in MS patient CSF. These contradictory results could be explained by the fact that kynurenines were measured in different phases of the disease: low KYNA levels were found in the acute relapse phase [76], while high KYNA levels are present in the relapse [77,78], and also in the remission phases of this disease [79].

Increased QA levels and a higher QA/KYN ratio were found in RRMS patients in the relapse phase, while lower TRP and KYNA levels were found in the secondary progressive phase of MS (SPMS). However, all metabolite concentrations were elevated in primary progressive MS (PPMS) [79]. Therefore, KP profile during disease progression consists of its own activation in the active phase of MS, which leads to an increase in KYNA levels and QA production, while these KYNA levels decrease in the progressive phase of the disease, converting KP metabolites into neurotoxic molecules [63,76,78]. Nevertheless, the elevated KYNA concentration reported in RRMS may counteract the neurotoxicity induced by QA residues, which may represent a compensatory mechanism present in the initial phase of the disease, with a moderate correlation reported between the QA/KYNA ratio and MS severity [71]. In progressive MS with intermittent exacerbations (PRMS), kynurenine metabolites exhibit distinct patterns that reflect underlying pathological processes. Elevated KYN, 3-HK, and QA levels are associated with increased inflammation and neurotoxicity, whereas decreased KYNA levels contribute to reduced neuroprotection. Monitoring these metabolites could improve disease management and guide therapeutic interventions [71].

The results of metabolic profiling of MS patients show that the KYNA/QA ratio is able to determine the excitotoxic activity of KP and may represent a predictive model for MS subtype progression by assessing the levels of six possible predictive characteristic biomarkers of the disease: KYNA, QA, tryptophan, picolinic acid, fibroblast growth factor, and TNF-α (in order of relevance) with a sensitivity of up to 85–91%. According to this order of relevance, the metabolomic profile confirms that the highest KYNA levels are present in RRMS patients compared to healthy controls and also in patients with the progressive MS type. In contrast, the lowest KYNA concentrations were found in PPMS and SPMS patients [71].

### 7.3. Effects of the Metabolites of Kynurenine on Brain Cells

The mechanisms responsible for the uptake of TRP by microglia once this molecule crossed the BBB are not fully understood. The presence of LAT1 was detected in microglia and other brain cells, suggesting that it is involved in the mechanism of uptake of this amino acid by brain cells. However, it is possible that other mechanisms play a role in the development of MS, as the expression of LAT1 in brain cells is lower than in the BBB [80].

Once TRP passed the BBB in excess, it is internalized by microglia and macrophages from this inflammatory environment. The expression of enzymes of the KP gained importance, as macrophages show a high expression of these enzymes. It was therefore shown that macrophages make an important contribution to neuronal cytotoxicity [81]. As previously mentioned, 3-HK and QA are two intermediates of KP and were postulated as major contributors to neurodegeneration in MS [82,83,84].

Recently, KMO and its metabolite 3-HK were detected in astrocytes, microglia, and neurons, but its activity is different in these cells. The highest KMO activity was found in neurons compared to that observed in microglia. Even when KMO is present in astrocytes, this enzyme is not active [53]. Therefore, the cytotoxic effect of 3-HK is more evident in neurons, possibly due to the accumulation of this metabolite. In microglia, 3-HK is not only the biological precursor of QA, but also contributes to the development of the inflammatory brain environment. The literature on the role of 3-HK in macrophages is controversial. Aarsland et al. showed that the metabolism of 3-HK in human macrophages leads to the formation of QA [67]. In contrast, Chiarugi et al. performed in vitro assays with macrophages stimulated with INF-γ and found that 3-HK is released outside the cell [29].

The toxic effect of 3-HK is due to its autooxidation, which leads to an increase in reactive oxygen species (ROS) levels, especially H_2_O_2_, xanthommatin (XAN), and 4,6-dihydroxy Qaoline Qaone carboxylic acid (DHQCA) [85,86]. Activation of the inflammatory process could limit the autooxidation of 3-HK released by macrophages, but the process of autooxidation of this metabolite in the extracellular space consisting of an inflammatory environment of the brain needs further investigation [87]. However, it is hypothesized that 3-HK, when released by macrophages, can induce high levels of H_2_O_2_ in the extracellular environment, whereupon the increased ROS levels would rise and cause the widely reported lipid peroxidation and protein oxidation [88]. Other studies showed that H_2_O_2_ is not only involved in the activation of signaling pathways leading to cell death, but also plays a modulatory role in the cytoskeleton organization [89]. In addition, 3-HK can be transported into neurons by neutral amino acid transporters, but its high concentration in cells may also be favored by the degradation of KYN, which readily enters cells and is then degraded to 3-HK by KMO [69]. In cells, 3-HK generates H_2_O_2_ by autooxidation, but the presence of xanthine oxidase also contributes to the increase in these H_2_O_2_ levels [67]. Therefore, intracellular concentrations of H_2_O_2_ are generated in the cell, leading to DNA damage, mitochondrial dysfunction, as well as lipid and protein peroxidation, thus triggering the activation of death cell pathways, such as apoptosis [69,86].

A distinction must be made between the internal signaling pathways that QA activates in macrophages and microglia and the extracellular signaling pathways that this molecule activates. Within microglia, QA activates NF-kβ through the degradation of Ik-Bα, enabling the transcription of TNF-α [90]. When NF-κβ is activated, it regulates the expression of inflammatory cytokines, such as IL-6 and IL-8 [91,92], and controls the production of reactive nitrogen species (RNS). These combined events amplify the inflammatory and oxidative environment, increasing cell damage and thus exacerbating the neurodegeneration process [92,93].

Once QA is released from macrophages and microglia, it exerts a strong excitatory effect on NMDA receptors in neurons [83,94]. The sustained excitation of NMDA-dependent mechanisms in neurons elicits the deregulated entry of Ca^2+^ into cells [95]. Under controlled conditions, calcium ions play an important role in the transmission of depolarization and synaptic activity. Therefore, it is evident that their deregulation has functional consequences that affect the viability of neurons [96]. Activation of the NMDA receptor by QA is characterized by an increase in the NO radical [97], which can generate radicals associated with DNA damage. This process activates enzymes such as poly-(ADP-ribose) polymerase-1, resulting in NAD^+^ and ATP loss that impairs mitochondrial permeability and increases superoxide production [98,99,100]. The overall oxidizing environment within the neuron finally leads to lipid peroxidation, which in turn leads to cell damage.

### 7.4. Activation of Intracellular Pathways by Inflammatory Process

One of the most important enzymes for the formation of KP metabolites is IDO, which is abundant in various tissues [101]. Although the presence of IDO was characterized in the brain, its activity was found to be low under basal conditions compared to other tissues, such as lung and intestine. This lower brain activity is important because IDO-induced tryptophan deficiency can have negative consequences leading to depression [102,103]. However, under certain conditions, increased brain IDO activity can be observed, directly contributing to an increase in tryptophan metabolites and exerting a neurotoxic effect in this tissue [103,104]. The localization of IDO was well characterized by in vitro assays in the experimental autoimmune encephalomyelitis model, the most commonly used model to understand the mechanisms observed in MS [84]. Using this model, IDO was shown to be present in both microglia and macrophages, demonstrating their involvement in the modulation of KP and the production of its metabolites and highlighting the involvement of these cell types in the development of excitotoxicity exerted by KP in MS [32,105].

Although the mechanisms by which MS induces inflammation in patients are not fully understood, the release of IFN-γ, predominantly by Th1 cells, increases the expression of IDO in microglia and macrophages [106,107,108,109]. Some studies show that following the binding of INF-γ to its receptor on the microglial membrane, the JAK/STAT1 pathway is activated, with the tyrosine kinase Janus kinase 2 (JAK2), which is now able to phosphorylate STAT1 and induce its dimerization, favoring its translocation to the nucleus where it recognizes elements of the INF-γ-responsive gene, thus increasing the expression of IDO in these cells [81,110,111]. Another mechanism by which INF-γ increases IDO expression is through the PI3K signaling pathway, which is not only involved in cell proliferation and survival mechanisms, but also modulates STAT1 activity. Interestingly, when the PI3K signaling pathway is inhibited in microglia, the induction of IDO expression is also negatively regulated. Furthermore, IDO expression is enhanced by the PI3K signaling pathway in the presence of IL-4, but the mechanisms involved are still unclear [112]. Additionally, the binding of INF-γ to its receptor on microglia activates the transcription of chemokines to recruit T cells, the generation of IL-7, IL-15, and IL-1ab, and the expression of several genes related to the major histocompatibility complex, all of which create a microenvironment that triggers neurodegenerative processes through the activation of microglia [112,113].

**Figure 4:**
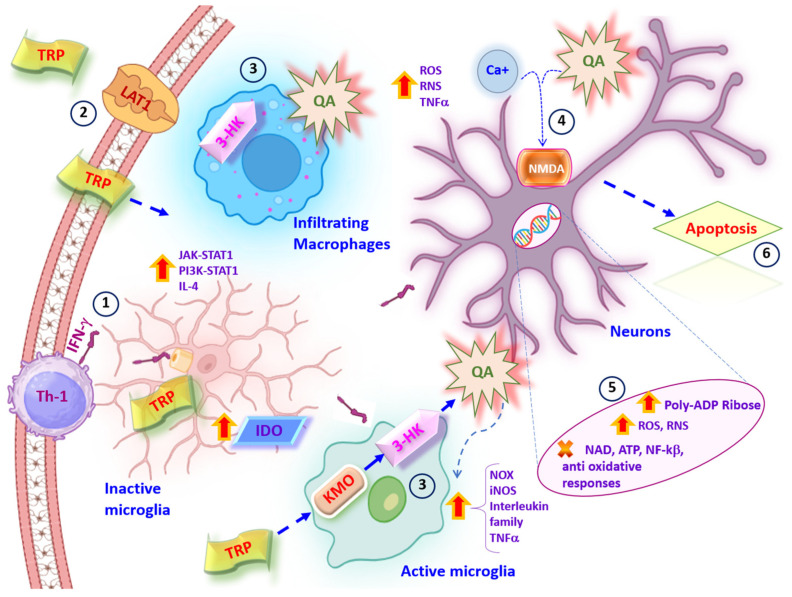
Activation of intracellular signaling pathways by inflammatory processes. In MS, tryptophan metabolism is deregulated and some cellular signaling pathways, such as JAK/STAT1 and AKT/STAT1/IL-4, are activated. (1) During inflammation, Th-1 cells release INFγ, which in turn increases the expression of IDO in microglia and infiltrating macrophages. (2) The increase in IDO enables the degradation of tryptophan, which crosses the BBB mainly through LAT1 transporters or due to changes in BBB membrane permeability. (3) Activation of KMO in microglia and infiltrating macrophages produces 3-HK and QA, which leads to an increase in RNS and ROS levels and allows the expression of TNF-α and pro-inflammatory interleukins, exacerbating the inflammatory environment. (4) QA negatively affects NMDA receptors and Ca+ uptake in neurons, (5) promotes the increase in reactive species levels and poly-(ADP-ribose) polymerase 1 activity, leading to depletion of NAD^+^, NF-kβ, and ATP, (6) and triggers cell death by apoptosis. Vertical red-yellow arrows indicate elevated levels of the indicated interleukins, enzymes, KP metabolites, signaling pathways factors and oxidative stress molecules.

As mentioned above, under normal conditions, TRP can cross the BBB towards the extracellular fluid via LAT1 receptors, which are abundant in the endothelium that forms this biological barrier [114,115,116]. In some experimental models of inflammation with TNF-α or lipoporisaccharide, it was shown that an increase in the expression of LAT1 in brain endothelial cells correlates with an increased uptake of TRP, thus increasing the availability of this amino acid in the CSF and allowing its uptake by microglia or active macrophages (Figure 4) [117]. However, other mechanisms may also contribute to the increase in CSF TRP levels, as inflammatory cytokines alter the permeability of the BBB and influence the role of cross-talk complex proteins present in the endothelial cells of this brain barrier. Nonetheless, further studies are needed to support these hypotheses [118].

### 7.5. Kynurenine’s Receptors on Inflammatory Cells

During the inflammatory response triggered by MS, immune cells play a crucial role in initiating and maintaining the unregulated inflammatory state as infiltrating macrophages and activated microglia are directed and colocalized around the nerve lesions. In addition, other immune cells, such as monocytes, are activated as a result of high levels of inflammatory cytokines that damage oligodendrocytes and upregulate KP enzymes expression, leading to increased production of neurotoxic QA and thus contributing to MS pathology [72,119].

KYN produced by antigen-presenting cells (APCs) is the paracrine signal for T cells that do not constitutively express IDO1 to take up KYN by active transport via the large neutral amino acid transporter (LAT-1) [120]. KYN also induces the expression of forkhead box P3 (FoxP3) for T-Reg cell differentiation and function, but simultaneously inhibits retinoic acid receptor-related orphan receptor-γt (RORγt), which promotes the differentiation of proinflammatory Th17 cells [121]. KYN can also indirectly modulate T-cell differentiation by modulating APC activities [122]. In contrast, KYNA can exert important anti-inflammatory effects, such as the inhibition of the differentiation of CD4+ T cells to the Th17 phenotype and suppressing the release of TNF, IL-4, and IL-23 from activated monocytes [123,124].

### 7.6. T-Reg Responses to Kynurenine Signaling

Kynurenines play a crucial role in the regulation of immune responses mediated by T cells. In particular, L-kynurenine inhibits antigen-specific T cell proliferation and induces apoptosis. This leads to a shift towards Th2 dominance and promotes anti-inflammatory responses. In addition, kynurenines induce the formation of FoxP3+ regulatory T cells, a specific subset of regulatory T cells (T-regs) characterized by the expression of FoxP3 by dendritic cells and suppress both Th1 and Th2 cells, restoring immune balance [125].

Kynurenines act as ligands for specific receptors, including glutamate receptors and the aryl hydrocarbon receptor, which are present on various immune cells, including T-regs. These cells play a critical role in maintaining immune tolerance and suppressing autoimmune responses. Kynurenines were shown to modulate the function and plasticity of T-regs, suggesting a possible mechanism by which these molecules influence the progression of MS [126].

Recent studies revealed that kynurenines can promote the expansion and functional activity of T-reg in MS models, leading to suppression of the autoimmune response and attenuation of neurodegenerative inflammation. In addition, it was suggested that the activation of aryl hydrocarbon receptor by kynurenines may induce the differentiation of naïve T cells into T-reg, enhancing their immunoregulatory role in the context of MS [127].

However, the relationship between kynurenines and T-reg in MS is complex and not yet fully understood. Further research is needed to elucidate the exact mechanisms by which kynurenines modulate T-reg responses and to determine their effects on MS pathogenesis. In addition, the identification of novel drugs that selectively modulate kynurenine signaling may offer new therapeutic strategies for MS and other autoimmune diseases [128].

## 8. Pharmacological Modulation of Kynurenine-Inflammation Axis

As mentioned above, different changes in the concentrations of several metabolites of KP (both in CSF and serum) were documented consistently in MS patients at different stages and clinical variants of the disease, as well as in models of experimental allergic encephalomyelitis (EAE) [61]. This undoubtedly suggests an involvement of KP in the pathophysiology of the disease. However, the final interpretation of whether these changes represent a main damaging mechanism of the disease, a compensatory response, or only an epiphenomenon remains to be determined in subsequent studies [26]. In this sense, knowledge of the involvement of KP in MS has essentially two potential clinical applications: (1) kynurenines serve as biomarkers of disease activity, markers of therapeutic responses or markers that allow the identification of clinical endophenotypes of the disease, which in turn allow the development of more individualized and effective treatments; and (2) they provide new therapeutic targets; it is precisely these types of therapeutic trials based on KP modulation that may potentially further support the role of KP in MS [129].

One of the most studied therapeutic targets in the literature is the modulation of the IDO enzyme [128]. Experimental studies showed that in IDO-knockout mice, Th1 and Th17 responses are greatly increased, and exacerbate brain injury in the EAE model. In contrast, exogenous administration of 3-HAA produced the opposite response by decreasing Th1 and Th17 activation and ameliorating brain and behavioral changes in the EAE model [130]. Similarly, several studies showed that the use of different IDO inhibitors worsens consistently the changes in EAE [131]. A recent study showed that the effects of EAE were very effectively suppressed by using different IDO agonists in addition to the inhibition of the enzymes KAT II and 3-hydroxyanthranilic acid dioxygenase and that no improvement occurred when the different IDO stimulation approaches were used separately [132]. In contrast, a recent study analyzed two KP modulation strategies: inhibition of IDO-1 with 1-methyl-tryptophan and inhibition of KMO in chronic stage EAE (aiming to model the progressive forms of MS). The study showed that both strategies improved the disease, however, the effect was more pronounced with KMO inhibitors [133]. On the other hand, previous evidence showed that melatonin has beneficial effects on EAE through multiple mechanisms [134,135,136]; as well as the observation that melatonin effectively inhibits the activity of TDO and IDO-1 [137]. In a more recent study, different doses of melatonin were tested in the EAE model, and both morphological and behavioral improvements were observed, but as an additional outcome, a reduction in the expression of IDO-1, a reduction in the aryl hydrocarbon receptor, and also an inhibition of the enzyme nicotinamide N-methyltransferase was also observed, suggesting that KP modulation may be an additional mechanism by which melatonin produces its beneficial effects in the EAE model [137].

Another type of therapeutic strategy that was investigated is the use of KP precursors. In a recent study, the administration L-KYN was explored in two types of approaches, a prophylactic approach in which treatment with L-Kyn was administered simultaneously with the protocol to induce EAE, and a therapeutic approach in which L-KYN was administered once the experimental model was on behavioral scale 2. Prophylactic administration of L-KYN resulted in a significant reduction in behavioral changes, while therapeutic administration resulted in a small and delayed improvement compared to prophylactic administration [138].

Finally, the drug laquinimod, an oral immunomodulator that was recently tested in MS patients, should be mentioned in this section. Structurally, laquinimod is a quinoline-3-carboxamide that has a high structural similarity to kynurenic acid and was shown experimentally to have several immunomodulatory and anti-inflammatory mechanisms: it reduces the expression of genes of MHC class II molecules; it decreases the expression of various genes involved in the activation of B and T cells; it reduces the release of proinflammatory cytokines (TNFα and IL-1); it inhibits the migration of T cells into the CNS; and it increases the release of anti-inflammatory cytokines (TGF-β and IL-4), among other mechanisms [61]. However, one of the drug features that perhaps attracted the most attention is that it leads to an increase in BNDF levels in different mice brain structures (such as striatum, lateral septal nucleus, nucleus accumbens, and cerebral cortex). Therefore, it is thought to have a neuroprotective effect, and it was documented that up to 76% of patients receiving this drug had an increase in serum BDNF [61,139]. Clinical trials with laquinimod in relapsing–remitting MS patients in phase III have so far shown a modest effect in reducing the number of relapses, the number of lesions on MRI and the rate of cerebral atrophy. However, precaution must be taken while applying this treatment, as the experimental models the incidence of malignancy appears to be increased; although no significant adverse effects were reported in clinical trials to date, warranting that its therapeutic benefits will be further explored in future studies [61,140].

### Drugs Repositioning for Multiple Sclerosis Treatment

Drug repositioning is a promising approach that is based in drugs already approved for other diseases and avoids the major obstacle of safety testing in drug development. Repositioning can significantly accelerate the availability of “new” therapies and leverage earlier research investments. However, in-depth biological knowledge is required to predict which approved drugs might be effective in another disease. Systematic literature reviews and genome-wide studies opened new opportunities by revealing new targets for existing drugs and discovering entirely new drug targets. MS is no exception and represents a challenge for researchers to identifying already approved drugs that could provide a potential benefit in the treatment of this neurodegenerative disease.

A systematic analysis of clinical data from 55 treatment options for progressive MS suggested R-α-lipoic acid and metformin as potential candidates [141]. Interestingly, α-lipoic acid is an antioxidant and a cofactor for the mitochondrial PHD enzyme, which links glycolysis and the TCA cycle [142]. As previously discussed, KP exerts important effects on cell metabolism and mitochondrial functions [143], so it is possible that α-lipoic acid and KP could indirectly influence each other. On the other hand, metformin, a known regulator of glucose metabolism, could also inhibit KP in brain tissue [144], underlining the need for further investigation in the context of MS.

A further systematic review identified six potential drugs for further research into MS treatment, including Ibudilast (an anti-inflammatory drug prescribed for asthma patients), Riluzole (an inhibitor of glutamate release), Amiloride (a diuretic), Pirfenidone (a TGF-β and TNF-α synthesis inhibitor used for pulmonary fibrosis), Fluoxetine (a serotonin reuptake inhibitor used as an antidepressant), and Oxcarbazepine (a sodium channel blocker used as an anticonvulsant) [145]. Pirfenidone inhibits KP in pulmonary fibrosis [146], and Fluoxetine is reported to inhibit KP in an animal model of lung cancer [147]. Evaluating their efficacy for MS treatment and their effects on the KP would be an interesting area of research.

Based on bulk and single-cell transcriptomic data from MS patients, two independent studies suggested targeting the PI3K-Akt signaling pathway as a promising approach. Chemokine signaling pathways, the AIM2 inflammasome, the SMAD2/3 pathway, and complement activation were also proposed as alternative repurposing targets for MS [148,149]. These signaling pathways could be inhibited with already approved drugs, such as Nemiralisib, Umbralisib, and the existing MS drug Mitoxantrone. Although the effect of such drugs on the KP was not studied, all of these signaling pathways are related to inflammatory responses and cell growth mechanisms in which the KP is directly involved [26,150].

Finally, the investigation of structural analogs of KYN and KYNA, such as Teriflunomide and Laquinimod, as previously discussed, suggests their use as potential MS therapies, considering that Teriflunomide is already an FDA-approved drug [26]. Overall, these examples highlight the potential of direct or indirect targeting of the KP for MS therapy.

## 9. Conclusions

Multiple sclerosis is a neurodegenerative disease whose etiology is not fully understood and whose prevalence is becoming a worrying public health problem in some countries, such as the USA, where around 400 cases per 100,000 inhabitants are reported, affecting mainly working people and therefore having a significant impact on healthcare costs. Currently, there is no specific diagnostic method for this disease, so the results obtained by MRI can provide information on the presence of typical demyelinating lesions, but already at advanced stages of the disease.

The multidisciplinary approach to MS allowed researchers to find a possible convergence point between the inflammatory response that occurs at different stages of the development of this neurological disease and the signals resulting from the activation of the kynurenine pathway. The uncontrolled uptake of tryptophan and its accumulation in the CNS leads to the production of cytotoxic KP metabolites that exacerbate the cellular damage triggered by an uncontrolled inflammatory response. Currently, the therapies recommended for these patients are aimed at controlling neuroinflammation to limit the damage caused by the inflammatory response with the consequent loss of neurons and progressive demyelination of axons, thus reducing the severity of the disease [39]. Similarly, some studies focused on the use of drugs structurally similar to KYN to modify the progression of MS, given the close relationship between some intermediates of the KP and factors such as NF-kβ, PPARγ and PGC-1 involved in the inflammatory response [151]. Therefore, the development of more potent and specific KP inhibitors in conjunction with the function of already approved drugs involved in other signaling pathways, proposed here as repurposing drugs, may help to find new and promising approaches to discover protective mechanisms against myelin sheath damage and neuronal death to eventually provide effective therapies for the treatment of MS patients and improve their quality of life. By carrying out this type of study, encouraging results could be achieved by using some drugs that are structurally similar to kynurenines with the aim to modify the progression of this disease [151].

On the other hand, more studies aimed at limiting the imbalance between the Th1/Th2 subpopulations and the regulatory T cells (Treg) responses, whose production of pro-inflammatory and anti-inflammatory interleukins, respectively, determines the severity and chronicity of MS, must also be carried out. In this sense, it was also reported that TNFα triggers neuronal death through a mechanism recently termed silencing of survival signals (SOSS) [152].

Recently, it was reported that 30 min of moderate aerobic exercise can reduce levels of inflammatory cytokines, such as IL-6, TNF-α, and TNF-γ, suggesting that exercise may be involved in the activation of IDO, the enzyme responsible for the conversion of TRP to KYN, which increases available tryptophan levels and favors an increase in serotonin production and a reduction in cytotoxic KYN levels [38,153].

Finding methods for timely clinical diagnosis of multiple sclerosis and proposing methodological approaches that make it possible to halt the progression of the disease, alleviate its symptoms or reverse its neurodegenerative effects remain an interesting challenge for researchers, with the aim of offering these patients better treatments and improve their quality of life.

## Data Availability

The data presented in this study are available on request from the corresponding authors.
